# The Prevalence of Psychotic Symptoms, Violent Ideation, and Disruptive Behavior in a Population With SARS-CoV-2 Infection: Preliminary Study

**DOI:** 10.2196/36444

**Published:** 2022-08-16

**Authors:** Sumra Bari, Nicole L Vike, Khrystyna Stetsiv, Sean Woodward, Shamal Lalvani, Leandros Stefanopoulos, Byoung Woo Kim, Nicos Maglaveras, Hans C Breiter, Aggelos K Katsaggelos

**Affiliations:** 1 Department of Psychiatry and Behavioral Sciences Northwestern University Chicago, IL United States; 2 Department of Electrical and Computer Engineering Northwestern University Evanston, IL United States; 3 Laboratory of Medical Informatics Aristotle University of Thessaloniki Thessaloniki Greece; 4 Laboratory of Neuroimaging and Genetics Division of Psychiatric Neuroscience, Massachusetts General Hospital Charlestown, MA United States

**Keywords:** COVID-19, paranoia, delusions, disruptive behavior, violent ideation, psychotic symptoms, pandemic, mental health, distress, stress, psychological health, psychosis, risk, machine learning

## Abstract

**Background:**

The COVID-19 disease results from infection by the SARS-CoV-2 virus to produce a range of mild to severe physical, neurological, and mental health symptoms. The COVID-19 pandemic has indirectly caused significant emotional distress, triggering the emergence of mental health symptoms in individuals who were not previously affected or exacerbating symptoms in those with existing mental health conditions. Emotional distress and certain mental health conditions can lead to violent ideation and disruptive behavior, including aggression, threatening acts, deliberate harm toward other people or animals, and inattention to or noncompliance with education or workplace rules. Of the many mental health conditions that can be associated with violent ideation and disruptive behavior, psychosis can evidence greater vulnerability to unpredictable changes and being at a greater risk for them. Individuals with psychosis can also be more susceptible to contracting COVID-19 disease.

**Objective:**

This study aimed to investigate whether violent ideation, disruptive behavior, or psychotic symptoms were more prevalent in a population with COVID-19 and did not precede the pandemic.

**Methods:**

In this preliminary study, we analyzed questionnaire responses from a population sample (N=366), received between the end of February 2021 and the start of March 2021 (1 year into the COVID-19 pandemic), regarding COVID-19 illness, violent ideation, disruptive behavior, and psychotic symptoms. Using the Wilcoxon rank sum test followed by multiple comparisons correction, we compared the self-reported frequency of these variables for 3 time windows related to the past 1 month, past 1 month to 1 year, and >1 year ago among the distributions of people who answered whether they tested positive or were diagnosed with COVID-19 by a clinician. We also used multivariable logistic regression with iterative resampling to investigate the relationship between these variables occurring >1 year ago (ie, before the pandemic) and the likelihood of contracting COVID-19.

**Results:**

We observed a significantly higher frequency of self-reported violent ideation, disruptive behavior, and psychotic symptoms, for all 3 time windows of people who tested positive or were diagnosed with COVID-19 by a clinician. Using multivariable logistic regression, we observed 72% to 94% model accuracy for an increased incidence of COVID-19 in participants who reported violent ideation, disruptive behavior, or psychotic symptoms >1 year ago.

**Conclusions:**

This preliminary study found that people who reported a test or clinician diagnosis of COVID-19 also reported higher frequencies of violent ideation, disruptive behavior, or psychotic symptoms across multiple time windows, indicating that they were not likely to be the result of COVID-19. In parallel, participants who reported these behaviors >1 year ago (ie, before the pandemic) were more likely to be diagnosed with COVID-19, suggesting that violent ideation, disruptive behavior, in addition to psychotic symptoms, were associated with COVID-19 with an approximately 70% to 90% likelihood.

## Introduction

### Background

The COVID-19 disease results from infection by the single-stranded RNA virus SARS-CoV-2. Physically, COVID-19 can produce a range of mild symptoms, including fever, cough, shortness of breath, fatigue, muscle aches, headache, and new loss of taste or smell [1], to severe symptoms requiring ventilation in an intensive care unit because of respiratory failure, septic shock, and multiple organ dysfunction [1]. In the context of mental function, COVID-19 has been linked to significant cognitive and attention deficits, brain fog, anxiety, depression, and sleep problems [2,3]—all of which can affect mental health and well-being.

The COVID-19 pandemic has burdened the mental health of both those infected with SARS-CoV-2 and those who have been living through the pandemic without infection. The pandemic has indirectly caused worldwide emotional distress that has triggered the development of mental conditions in persons not previously affected and exacerbated symptoms in those with existing conditions [4-7]. Numerous COVID-19 research reviews have reported the adverse psychological effects brought on by pandemic-related stressors, including posttraumatic stress symptoms, confusion, and anger [8]. The management of behavioral symptoms in patients with COVID-19 (eg, agitation) has become a unique challenge for health care workers in emergency departments [9]. COVID-19 can be particularly distressing to individuals with pre-existing mental health conditions such as autism spectrum disorders and other neurodevelopmental disorders, leading to more intense and frequent behavior problems, including disruptive behavior [10] or increased externalizing and aggressive behavior [11].

Many mental health conditions can lead to violent ideation and disruptive behavior (VIDB), which is defined by aggression, threatening acts, or deliberate harm toward other people or animals, as well as inattention and noncompliance in education or workplace settings [12,13]. For instance, people with substance use disorders [14,15], mania [16,17], psychosis [18,19], and personality disorders [20,21] can all evidence VIDB or be at an increased risk for adverse psychosocial outcomes during pandemic-related periods of stress. Individuals with mild to severe symptoms of psychosis (eg, delusions, paranoia, and hallucinations) have been reported to be susceptible to pandemic-related emotional distress [18,19]. The 12-month prevalence of psychosis is 3.89 to 4.03 per 1000 individuals, and the median lifetime prevalence is 7.49 per 1000 individuals [22]. Prior research has shown that people with psychosis are less likely to take precautionary measures such as receiving vaccination or isolating during the influenza pandemic [23], thereby increasing their potential risk for COVID-19 infection. As with autism spectrum disorder, individuals with psychosis can show greater vulnerability to unpredictable changes, such as COVID-19, and are thus at greater risk for VIDB [24,25].

### Objective

In this preliminary study, we assessed potential relationships among COVID-19 infection, VIDB, and symptoms related to psychosis, which may increase the risk of infection and VIDB. Psychotic symptoms and VIDB increase the potential for engagement in riskier-than-average behaviors, potentially including nonadherence to COVID-19 precautions. Individuals exhibiting VIDB and psychotic symptoms could thus be at a greater risk of contracting and spreading the virus. Although these associations are not directional, any findings between VIDB and psychotic symptoms with COVID-19 would permit hypothesis framing for two scenarios: (1) COVID-19 infection increases the likelihood of psychosis and VIDB or (2) psychotic symptoms and VIDB increase the possibility of COVID-19 infections. Other viral infections, including influenza and HIV, have led to similar hypotheses [26-34]. To do this, we analyzed questionnaire responses regarding COVID-19 history along with historical questions regarding VIDB and psychotic symptoms over 3 time windows in a small but representative internet sample that followed the US Census (ie, 300<n<500). The questionnaire was distributed between the end of February 2021 and the start of March 2021 and was timed to overlap with the onset of the COVID-19 pandemic 1 year earlier. We assessed the frequency of self-reported VIDB and symptoms of psychosis from the past 1 month, 1 month to 1 year ago, and >1 year ago among participants with and without self-reported COVID-19. Given the existing case reports of an increased incidence of VIDB and psychotic symptoms during the pandemic, we hypothesized that participants with COVID-19 would exhibit an increase in VIDB and psychotic symptoms. We also investigated the relationship between these behaviors occurring >1 year ago and the likelihood of contracting SARS-CoV-2 with the hypothesis that people with these behaviors were more likely to experience COVID-19 infection. We used multivariable logistic regression (MVLR) with iterative resampling to investigate how accurately VIDB and psychotic symptoms from before the pandemic discriminated between participants with and without self-reported COVID-19.

Altogether, the results of this study show that individuals with VIDB and psychotic symptoms have an increased risk for SARS-CoV-2, although long-term longitudinal data will be needed to assess causal relationships.

## Methods

### Participant Recruitment

Study participants were recruited by Gold Research Inc from multiple vendors. Gold Research vendors recruit the emails of willing participants in multiple ways. Some are recruited *by invitation only* from customer databases of large companies in revenue-sharing agreements, some are recruited from social media, some through direct mail, and others sign up voluntarily to participate in research studies in lieu of monetary or other incentives such as coupons for everyday household purchases. During recruitment, all survey respondents also go through a double opt-in process to indicate the types of research studies they would like to participate in, in addition to providing their profiles on different demographic attributes such as age, race, and sex. This information is then used to reflect representation against US Census metrics. In this process, respondents are also asked multiple test questions to screen out those providing random and illogical responses or showing flatline or speeder behavior. In addition to having cohort demographics balanced to meet the demographic criteria established by the US Census, Gold Research also oversampled 15% (7500/50,000) of the sample for mental health conditions. Gold Research reported that >50,000 respondents were contacted for questionnaire completion. They estimated that of the 50,000 participants, >37,500 (75%) either did not respond or said no. Of the remaining 25% (12,500/50,000) of participants who clicked on the survey link, >50% did not complete the questionnaire. Of the ≥6000 participants who completed the survey, those who did not clear the data integrity assessments were omitted to achieve the final number of completed surveys. Participants meeting quality assurance procedures (including completion of the survey) were studied, with a limit of 500-520 participants. All participants provided informed consent following oversight from the Northwestern University Institutional Review Board, and a double opt-in methodology was used for consenting (shown later in the following section).

**Figure 1:**
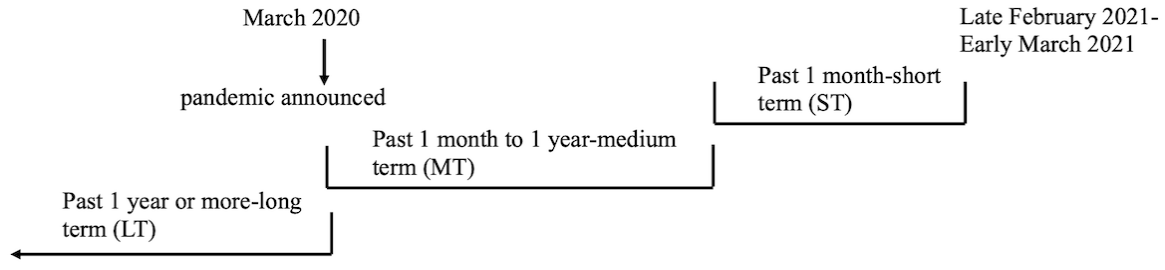
Timeline for survey data collection.

This study assessed multiple mental health conditions, including psychotic symptoms, violent ideation, and disruptive behavior. For this paper, we focused only on psychotic symptoms, violent ideation, and disruptive behavior. All participants provided informed consent following oversight from the Northwestern University Institutional Review Board, which reviewed and approved the project procedures, including the use of a double opt-in methodology for consenting. Participants were guaranteed anonymity and confidentiality, and the researchers did not possess any protected health information. Questionnaire responses were collected between the end of February 2021 and the beginning of March 2021, approximately 1 year after the official pandemic declaration in the United States (March 11, 2020) [35] (Figure 1). Data integrity assessment (see the *Data Quality Assurance* section) reduced the final sample size to 366 participants.

Participants reported their age, sex, ethnicity, handedness, annual household income, employment status, level of education, and years of schooling. A total of 506 participants (mean age 47, SD 15 years) completed the study by Gold Research Inc. After quality assurance, participants were found to be 57.9% (212/366) female, 68% (249/366) White, 82% (300/366) right-handed, 42.6% (156/366) employed full-time, and 28.7% (105/366) with some college education (mean years of schooling 13, SD 5 years), approximating the national averages for these measures. A complete summary of the demographic variables is provided in Table 1.

Participants were asked questions related to COVID-19, as shown in Textbox 1. They were asked to report whether they had ever had a positive COVID-19 test (yes or no; *test+*), whether they were ever diagnosed with COVID-19 by a medical professional (yes or no; *diagnosis*), and whether a family member or close friend had experienced serious symptoms or died of COVID-19 (yes or no; *family*). Of the 366 participants, 36 (9.8%) reported a positive COVID-19 *test+*, and 34 (9.3%) reported a COVID-19 *diagnosis*; 26 (7.1%) participants answered *yes* to both the COVID-19 *test+* and *diagnosis*. Of the 366 participants, 95 (26%) reported that a family member or close friend had serious symptoms or had died of COVID-19.

**Table 1:** Summary statistics of the demographic variables reported by the participants, including age, gender, ethnicity, handedness, annual household income, employment status, level of education, and years of schooling (N=366).

Variable		Values
**Age (years)**		
	Participants, n (%)	366 (100)
	Value, mean (SD; range)	46.67 (15.40; 18-70)
**Years of schooling**		
	Participants, n (%)	366 (100)
	Value, mean (SD; range)	13.29 (5.04; 1-30)
**Sex, n (%)**		
	Male	153 (41.8)
	Female	212 (57.9)
	Other	1 (0.3)
**Ethnicity, n (%)**		
	White	249 (68)
	African American	48 (13.1)
	Hispanic	29 (7.9)
	Asian American or Pacific Islander	13 (3.6)
	Native American or Alaskan Native	18 (4.9)
	Mixed race	3 (0.8)
	Other	3 (0.8)
	Prefer not to answer	3 (0.8)
**Handedness, n (%)**		
	Right	300 (82)
	Left	52 (14.2)
	Both	14 (3.8)
**Level of education, n (%)**		
	Some high school	6 (1.6)
	High school graduate	95 (26)
	Some college	105 (28.7)
	Bachelor degree	83 (22.7)
	Some graduate school	12 (3.3)
	Graduate degree	27 (7.4)
	Postgraduate or doctorate	38 (10.4)
**Employment status, n (%)**		
	Unemployed	58 (15.8)
	Full-time	156 (42.6)
	Part-time	41 (11.2)
	Self-employed	24 (6.6)
	>1 job	3 (0.8)
	Retired	61 (16.7)
	Other	23 (6.3)
**Annual household income (US $), n (%)**		
	<25,000	86 (23.5)
	25,000-50,000	92 (25.1)
	50,000-75,000	70 (19.1)
	75,000-100,000	47 (12.8)
	100,000-150,000	37 (10.1)
	150,000-300,000	26 (7.1)
	>300,000	8 (2.2)

**COVID-19–related questions from the survey and their abbreviations used in this study.**

**Abbreviations used and COVID-19 questions (yes or no)**

- Test+
  Have you ever tested positive for COVID-19?
- Diagnosis
  Have you ever been diagnosed with COVID-19 by a medical clinician?
- Family
  Has anyone in your family or group of friends had serious symptoms or died of COVID-19?

Power analysis for a 2-sample Wilcoxon rank sum test revealed an estimated power of >0.80 for the experimental conditions based on *test+* (sample size for *test+* was 36 and sample size for no *test+* was 330; α=.05) and an estimated power >0.9 for experimental conditions based on *diagnosis* (sample size for *diagnosis* was 34 and sample size for no *diagnosis* was 332; α=.05). This power suggests that we had statistically adequate sample sizes for the analysis that follows.

For initial recruitment, participants received the following communication:

Gold Research Inc., a national market research firm and its client, Northwestern University, request your participation in this study of emotional health. We will be evaluating how different emotions and experiences are connected and may relate to our emotional health. The information you provide will be kept confidential, coded to be anonymous so it cannot be connected back to you and will be used only for research purposes. Researchers will not be able to contact you or restudy you after this survey. We will not share your information with any other third party. We will also not use your information to identify you individually or use your responses to market or sell other services or products to you. As part of this effort, you will not be asked to provide any personal identifiers such as your name, email, phone number, address or social media handles. A unique identifier will be generated for you and each survey participant to enhance privacy. As part of the survey process, we will be able to tell if you completed the survey, but we will not be able to tell which answers were yours. For this study, we are going to ask you some questions about yourself and how much you like or dislike a set of pictures. You may discontinue this study at any time. We appreciate your help with this study, given the serious challenges facing many people regarding emotional health at this time. We thank you in advance.

1. Accept

2. Decline

If participants responded with “Accept,” they were sent a further communication with the following:

Thank you for participating in our survey. All responses during this survey are anonymous and confidential. We will be able to tell if you completed the survey, but we will not be able to tell which answers were yours. In this study, we aim to understand how different emotions and experiences relate to visual processing.

We are going to:

*Ask you some questions about yourself

*Have you rate how much you like or dislike a set of pictures

For this study, your identity is protected and your answers are anonymous and confidential. Press “Next” to proceed.

The survey would then begin if participants pressed “Next.”

### Ethics Approval

Participants were offered notice that Gold Research administered an emotional health questionnaire on behalf of Northwestern University, with the following phrasing: “We will be evaluating how different emotions and experiences are connected and may relate to our emotional health.” The complete text related to the solicitation, study description, and opt-in procedures can be found in the *Methods under Participant Recruitment*. All participants provided informed consent following oversight from the Northwestern University Institutional Review Board (approval number STU00213665), which reviewed and approved the project proposal. Participants were guaranteed anonymity and confidentiality, and the researchers did not possess any protected health information.

### Survey Questions and Scoring

**Figure 2:**
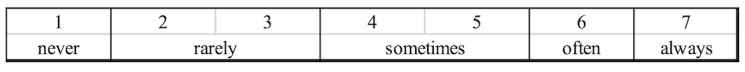
The Likert scale on which participants rated the questions.

The survey comprised several blocks of questions from existing questionnaires on depression, anxiety, suicidality, addiction, psychosis, VIDB, and COVID-19 infection, in addition to demographic and historical diagnoses of mental health disorders. A picture-rating task was also administered; however, it is not the focus of this study. This study assessed the relationships among psychotic symptoms, VIDB, and COVID-19 infection. Questions about VIDB and 4 positive psychotic symptoms were taken from the behavioral neurology screening questions in the Massachusetts General Hospital Subjective Question (MGH SQ) Screener from the Phenotype Genotype Project in Addiction and Mood Disorder [36]. The MGH SQ has been used in several studies [37-43]. Of the 13 questions used here, 4 were related to positive psychotic symptoms (auditory and visual hallucinations, paranoia, and delusions), which had originally been adapted to the MGH SQ from a clinical textbook on emergency psychiatry (Textbox 2, Psych1-Psych4) [44]. A total of 9 other questions were related to VIDB (eg, wanting to hurt others, prior attempts to hurt others or animals, wanting to start fires, being disruptive, or breaking rules) and were similarly adapted to the MGH SQ using the same clinical textbook on emergency psychiatry (Textbox 2, Disruption1-Disruption9) [44]. Participants rated the questions based on how often they experienced these behaviors as follows: (1) *past ≥1 year* (long term), (2) *past 1 month to 1 year* (medium term), *and* (3) *past 1 month* (short term; Figure 1 provides the timeline) on a 1 to 7 Likert scale (1=never; 2-3=rarely; 4-5=sometimes; 6=often; 7=always; Figure 2). The data were collected regarding psychotic symptoms and VIDB from the past 1 month (short term) and between 1 month and 1 year (medium term) to assess and relate these symptoms and behaviors during the recent past to COVID-19 infection. We chose a 1-month cutoff to obtain the most recent history, similar to what is done for timeline follow-back methods with substance use or menstrual cycle assessments. The data from >1 year ago (long term) were collected to assess these behaviors before the pandemic and to determine whether a history of psychosis and VIDB before the pandemic influenced a person’s likelihood of COVID-19 infection. This would provide insight into the participants’ current state and how COVID-19 infection or the pandemic, in general, has affected the general population. All 13 questions are listed in Textbox 2 as they appeared in the survey.

**The 13 questions from the larger survey related to psychosis and violent ideation and disruptive behavior that was used in this study for 3 time windows (long term, medium term, and short term) for which participants answered each question.**

**Questions and time windows**

- Psych1-LT
  Hallucinations/Hearing voices others cannot past ≥1 year
- Psych1-MT
  Hallucinations/Hearing voices others cannot past 1 month to 1 year
- Psych1-ST
  Hallucinations/Hearing voices others cannot past 1 month
- Psych2-LT
  Hallucinations/Seeing things others cannot see past ≥1 year
- Psych 2-MT
  Hallucinations/Seeing things others cannot see past 1 month to 1 year
- Psych2-ST
  Hallucinations/Seeing things others cannot see past 1 month
- Psych3-LT
  Worries that others are out to get you or to get people close to you (these might resemble paranoia) past ≥1 year
- Psych 3-MT
  Worries that others are out to get you or to get people close to you (these might resemble paranoia) past 1 month to 1 year
- Psych3-ST
  Worries that others are out to get you or to get people close to you (these might resemble paranoia) past 1 month
- Psych4-LT
  Having one or more unique beliefs or impressions that are strong despite being contradicted by others (these might resemble delusions) past ≥1 year
- Psych 4-MT
  Having one or more unique belief or impression that is strong despite being contradicted by others (these might resemble delusions) past 1 month to 1 year
- Psych4-ST
  Having one or more unique belief or impression that is strong despite being contradicted by others (these might resemble delusions) past 1 month
- Disruption1-LT
  Wanting to hurt others past ≥1 year
- Disruption 1-MT
  Wanting to hurt others past 1 month to 1 year
- Disruption1-ST
  Wanting to hurt others past 1 month
- Disruption2-LT
  Prior attempts at hurting others past ≥1 year
- Disruption 2-MT
  Prior attempts at hurting others past 1 year to 1 month
- Disruption2-ST
  Prior attempts at hurting others past 1 month
- Disruption3-LT
  Having a plan for not hurting others when these feelings arise past ≥1 year
- Disruption3-MT
  Having a plan for not hurting others when these feelings arise past 1 year to 1 month
- Disruption3-ST
  Having a plan for not hurting others when these feelings arise past 1 month
- Disruption4-LT
  Prior attempts at hurting insects or small animals past ≥1 year
- Disruption4-MT
  Prior attempts at hurting insects or small animals past 1 year to 1 month
- Disruption4-ST
  Prior attempts at hurting insects or small animals past 1 month
- Disruption5-LT
  Intrusive thoughts that lead you to repetitive actions past ≥1 year
- Disruption5-MT
  Intrusive thoughts that lead you to repetitive actions past 1 year to 1 month
- Disruption5-ST
  Intrusive thoughts that lead you to repetitive actions past 1 month
- Disruption6-LT
  Desire to start fires past ≥1 year
- Disruption6-MT
  Desire to start fires past 1 year to 1 month
- Disruption6-ST
  Desire to start fires past 1 month
- Disruption7-LT
  Being disruptive in a social environment (eg, at school or elsewhere) past ≥1 year
- Disruption7-MT
  Being disruptive in a social environment (eg, at school or elsewhere) past 1 year to 1 month
- Disruption7-ST
  Being disruptive in a social environment (eg, at school or elsewhere) past 1 month
- Disruption8-LT
  Attention problems past ≥1 year
- Disruption8-MT
  Attention problems past 1 year to 1 month
- Disruption8-ST
  Attention problems past 1 month
- Disruption9-LT
  Breaking rules at school or elsewhere past ≥1 year
- Disruption9-MT
  Breaking rules at school or elsewhere past 1 year to 1 month
- Disruption9-ST
  Breaking rules at school or elsewhere past 1 month

### Data Quality Assurance

Data quality used 4 exclusion criteria: (1) participants with the same responses throughout any section of the questionnaire (eg, “1” for all questions), (2) participants indicating they had been diagnosed by a clinician with ≥10 illnesses, (3) participants with minimal variance in a picture-rating task (all pictures were rated the same or varied only by 1 point; data not described here), (4) participants reporting inconsistent education level and years of education *and* participants who completed the questionnaire in <500 seconds. From these procedures, 366 participants were cleared for statistical analysis.

### Statistical Analyses

#### Analysis of Demographics and Survey Questions by Self-reported COVID-19 Infection

Demographic variables (Table 1) and survey question scores for all questions were divided into 2 distributions based on the *yes* and *no* responses from participants for each of the COVID-19 questions: *test+*, *diagnosis*, and *family*. Distributions of the demographic variables and survey question scores were assessed for differences using the Wilcoxon rank sum test [45]. Significant categorical demographic variables were further assessed for distribution equality using the Kolmogorov-Smirnov test (α=.05) [46]. The resulting *P* values for the questions *Psych1-LT*, *Psych1-MT*, *Psych1-ST*, and *Psych2-LT* to *Psych4-ST* were corrected for multiple comparisons using the Benjamini-Hochberg procedure [47] (reported as q[FDR]) for each COVID-19 question. The same procedure was repeated for questions *Disruption1-LT* to *Disruption9-ST*. The normalized test statistic from the Wilcoxon rank sum test and q(FDR) are reported. Box plots were generated to display the yes or no distribution for all survey questions and are presented in the Multimedia Appendix 1.

#### MVLR and Iterative Resampling: Using Demographics and Survey Questions to Model COVID-19 Likelihood

MVLR was performed with the goal of modeling COVID-19 likelihood. The aim was to determine how psychotic symptoms and VIDB from before the pandemic made people more vulnerable to COVID-19 infection during the pandemic. The set of *past ≥1 year (long term)* survey responses for the psychotic symptoms and VIDB, which significantly differed (q[FDR]) between those responding yes/no to *test+*, *diagnosis*, and *family* questions for COVID-19, were used as *independent variables*. The demographic variables with significant differences across yes or no responses for COVID-19 *test+*, *diagnosis*, or *family* (q[FDR]<0.05; Wilcoxon rank sum test) were used as *covariates* in the MVLR analyses. The COVID-19 question responses (yes=1 and no=0) were the binary *dependent variables*. However, the percentage of participants with a positive self-reported COVID-19 *test+* or *diagnosis* (yes=1; 9% to 10%) was much lower than the percentage of those without COVID-19 *test+* or *diagnosis* (no=0; approximately 90%), implying a class imbalance that could lead to model overfitting. To avoid overfitting the MVLR model to the majority class, data in the majority class (ie, participants in the COVID-19 *test+* no and *diagnosis* no groups) were randomly downsampled to match the sample size of those self-reporting COVID-19 (yes=0; 36/366, 9.8% for *test+* model, and 34/366, 9.3% for *diagnosis* model). Downsampling was iterated 1000 times, and MVLR was run at each iteration for the downsampled data to obtain the model accuracy, root mean square error (RMSE), and mean absolute error (MAE) of the model. The model accuracy was computed by comparing the number of times the model correctly determined the binary outcome divided by the size of the downsampled data. The average accuracy, SD of the accuracy, average RMSE, and average MAE across all iterations are reported.

## Results

### Overview

The demographic variables and the frequency of self-reported symptoms of psychosis and VIDB from the 3 time intervals were assessed for participants with and without self-reported COVID-19. For this assessment, we used a Wilcoxon rank sum test followed by multiple comparisons correction. The relationship between these behaviors occurring >1 year ago and the likelihood of contracting COVID-19 was investigated using MVLR with iterative resampling. Namely, we asked whether symptoms from >1 year ago could predict COVID-19 infection.

### Demographic Variables and Survey Question Responses Varied by Self-reported COVID-19

In summary, of the 366 participants, 36 (9.8%) reported a positive COVID-19 *test+*, and 34 (9.3%) reported a COVID-19 *diagnosis*; 26 (7.1%) participants answered yes to both the COVID-19 *test+* and *diagnosis*. Of the 366 participants, 95 (26%) reported that a family member or close friend had serious COVID-19–related symptoms or died of COVID-19. We found that participants who self-reported yes to contracting COVID-19 were middle-aged and belonged to the higher-income and higher education groups. Participants who self-reported yes to contracting COVID-19 also reported a higher frequency of psychotic symptoms and VIDB across all 3 time windows of the past 1 month, 1 month to 1 year ago, >1 year ago.

**Figure 3:**
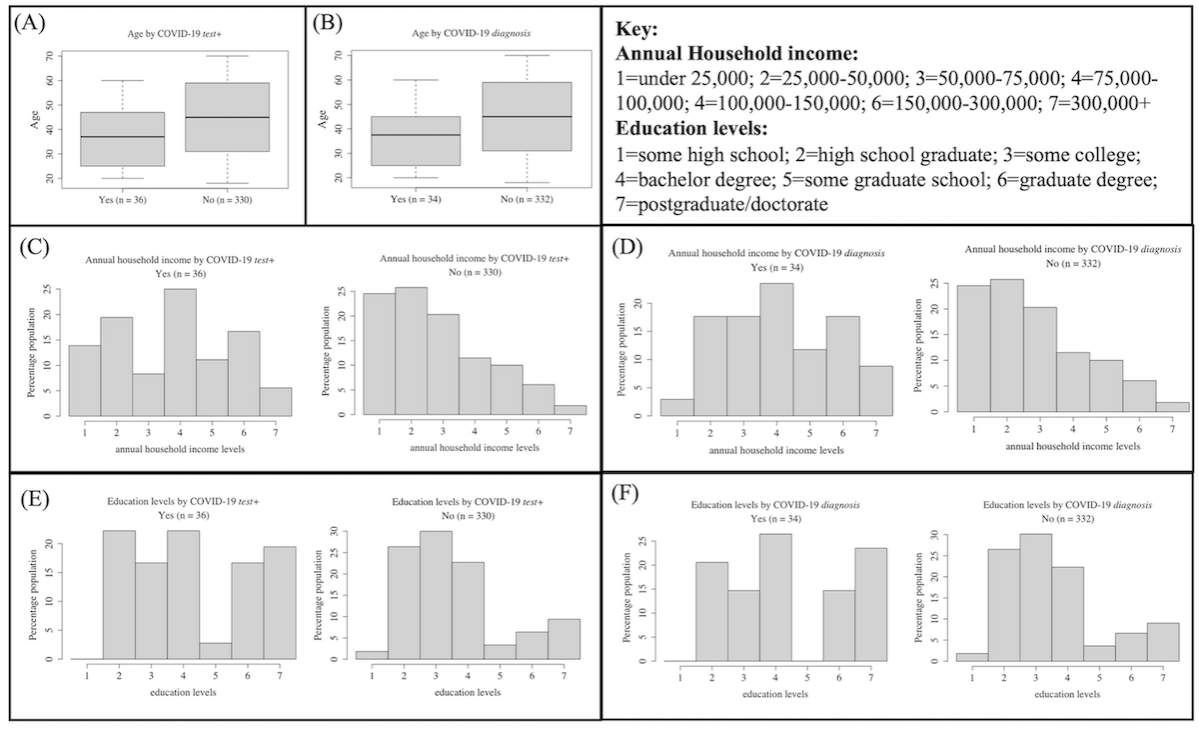
Box plots for distributions of age for participants who answered yes or no for (A) COVID-19 test+ and (B) COVID-19 diagnosis. Histograms for distributions of annual household income (US dollars) for participants who answered yes or no for (C) COVID-19 test+ and (D) COVID-19 diagnosis. Histograms for distributions of education for participants who answered yes or no for (E) COVID-19 test+ and (F) COVID-19 diagnosis.

Specifically, age and income significantly varied by *test+*, and age, income, and education level significantly varied by *diagnosis* (all q[FDR]<0.05; Wilcoxon rank sum test; Table 2). Participants who responded yes to *test+* and *diagnosis* were, on average, younger than those who responded no (Table 3; Figure 3A and 3B). Specifically, middle-aged adults more frequently reported yes to *test+* (IQR 25-47, median 37 years) and *diagnosis* (IQR 25-45, median 37.5 years) than those responding no (IQR 31-59, median 45). Participants responding no to *test+* or *diagnosis* showed a distribution with a higher percentage of low-income levels than those with COVID-19 (*P*=.004; *P*=.001; Kolmogorov-Smirnov test; Figure 3C and 3D). Participants responding yes to *test+* or *diagnosis* exhibited a bimodal distribution of education level, whereas the distribution for NO responses was skewed left (*P*=.04; *P*=.01; Kolmogorov-Smirnov test; Figure 3E and 3F) indicating a higher percentage of low education levels. None of the demographic variables exhibited significant differences based on *family* COVID-19 questions.

Furthermore, the survey questions for which responses significantly varied (q[FDR]<0.05; Wilcoxon rank sum test) by COVID-19 status (*test+*, *diagnosis*, and *family*) have q(FDR) highlighted in Tables 4 and 5. All 4 questions related to psychotic symptoms showed significant differences in medians across the yes and no distributions with respect to *test+* and *diagnosis for COVID-19* across the three time windows: (1) *past ≥1 year (long term),* (2) *past 1 month to 1 year (medium term), and* (3) *past 1 month (short term*; Table 4). All 9 questions related to VIDB also showed significantly different medians across the yes and no distributions with respect to *test+* and *diagnosis for COVID-19* across the 3 time windows (Table 5). There were no significant response differences across the 13 psychotic symptoms and VIDB questions relative to the COVID-19 *family* questions. Box plots based on *test+* and *diagnosis* for yes distribution had higher scores than for those who answered no for all survey questions, as presented in the Multimedia Appendix 1.

**Table 2:** List of the demographic variables that significantly differed by COVID-19 questions (Wilcoxon rank sum test)^a^.

Demographic	COVID-19 questions	*P* value	q(FDR)
Age	Test+^b^	.006	0.024^b^
Age	Diagnosed^b^	.004	0.02^b^
Employment	Diagnosed	.04	0.20
Income	Test+^b^	.004	0.02 ^b^
Income	Diagnosed^b^	<.001	<0.001^b^
Education level	Test+	.02	0.08
Education level	Diagnosed^b^	.009	0.04^b^

^a^Uncorrected **P* values* and *q(FDR)* are reported.

^b^Demographic variables with *q(FDR)* <.05.

**Table 3:** List of demographic variables that significantly differ by Kolmogorov-Smirnov test (*P*<.05).

Demographic	COVID-19 questions	*P* value (yes>no)	*P* value (no>yes)
Income	Test+	.004	>.99
Income	Diagnosis	.001	>.99
Education level	Test+	.64	.04
Education level	Diagnosis	.48	.01

**Table 4:** Survey questions related to psychosis assessed by COVID-19 status (test+, diagnosis, and family) using the Wilcoxon rank sum test.

Questions		COVID-19 questions							
		Test+			Diagnosis			Family	
		Test statistic	q(FDR)	Test statistic		q(FDR)	Test statistic		q(FDR)
**Hallucinations/Hearing voices others cannot**									
	Past ≥1 year	3.50	*6.21×10* ^−4^	4.78		*2.33×10* ^−6^	0.42		0.95
	Past 1 month to 1 year	3.51	*6.21×10* ^−4^	4.93		*1.21×10* ^−6^	0.06		0.95
	Past 1 month	4.17	*1.42×10* ^−4^	5.56		*7.88×10* ^−8^	0.08		0.95
**Hallucinations/Seeing things others cannot see**									
	Past 1 ≥year	3.61	*5.30×10* ^−4^	4.94		*1.21×10* ^−6^	0.11		0.95
	Past 1 month to 1 year	4.10	*1.42×10* ^−4^	5.92		*1.90×10* ^−8^	0.24		0.95
	Past 1 month	4.07	*1.42×10* ^−4^	5.96		*1.90×10* ^−8^	0.26		0.95
**Worries that others are out to get you, or to get people close to you (these might resemble paranoia)**									
	Past ≥1 year	3.82	*3.25×10* ^−4^	3.84		*1.24×10* ^−4^	1.09		0.95
	Past 1 month to 1 year	3.29	*1.08×10* ^−3^	4.27		*2.34×10* ^−5^	0.38		0.95
	Past 1 month	2.78	*5.51×10* ^−3^	3.88		*1.14×10* ^−4^	0.18		0.95
**Having one or more unique beliefs or impressions that are strong despite being contradicted by others (these might resemble delusions)**									
	Past ≥1 year	3.44	*6.95×10* ^−4^	5.39		*1.44×10* ^−7^	0.44		0.95
	Past 1 month to 1 year	3.66	*5.05×10* ^−4^	5.50		*8.99×10* ^−8^	0.67		0.95
	Past 1 month	4.16	*1.42×10* ^−4^	5.86		*1.90×10* ^−8^	0.46		0.95

**Table 5:** Survey questions related to violent ideation and suicidal behavior assessed by COVID-19 status (test+, diagnosis, and family) using the Wilcoxon rank sum test.

Questions		COVID-19 questions							
		*Test+*			*Diagnosis*			*Family*	
		Test statistic	q(FDR)	Test statistic		q(FDR)	Test statistic		q(FDR)
**Wanting to hurt others**									
	Past ≥1 year	4.13	*1.62×10* ^−4^	5.33		*3.88×10* ^−7^	0.61		0.72
	Past 1 month to 1 year	4.34	*1.42×10* ^−4^	5.35		*3.88×10* ^−7^	1.07		0.67
	Past 1 month	3.80	*3.19×10* ^−4^	4.77		*3.09×10* ^−6^	0.55		0.72
**Prior attempts at hurting others**									
	Past 1 ≥year	4.18	*1.58×10* ^−4^	4.90		*1.83×10* ^−6^	0.34		0.83
	Past 1 month to 1 year	3.77	*3.19×10* ^−4^	4.98		*1.39×10* ^−6^	0.81		0.67
	Past 1 month	4.01	*2.33×10* ^−4^	5.35		*3.88×10* ^−7^	0.65		0.72
**Having a plan for not hurting others when these feelings arise**									
	Past ≥1 year	2.21	*2.84×10* ^−2^	4.26		*2.48×10* ^−5^	1.16		0.67
	Past 1 month to 1 year	2.76	*6.43×10* ^−3^	4.10		*4.24×10* ^−5^	1.46		0.55
	Past 1 month	2.75	*6.45×10* ^−3^	4.20		*3.01×10* ^−5^	1.53		0.55
**Prior attempts at hurting insects or small animals**									
	Past ≥1 year	3.75	*3.24×10* ^−4^	5.33		*3.88×10* ^−7^	0.58		0.72
	Past 1 month to 1 year	3.87	*3.15×10* ^−4^	5.47		*3.88×10* ^−7^	0.87		0.67
	Past 1 month	3.85	*3.15×10* ^−4^	5.12		*7.32×10* ^−7^	1.43		0.55
**Intrusive thoughts that lead you to repetitive actions**									
	Past ≥1 year	3.40	*9.65×10* ^−4^	4.69		*3.80×10* ^−6^	0.26		0.86
	Past 1 month to 1 year	3.31	*1.27×10* ^−3^	4.53		*8.00×10* ^−6^	0.14		0.92
	Past 1 month	3.82	*3.19×10* ^−4^	5.15		*7.01×10* ^−7^	0.08		0.93
	**Desire to start fires**								
	Past ≥1 year	4.28	*1.42×10* ^−4^	5.18		*6.98×10* ^−7^	0.84		0.67
	Past 1 month to 1 year	4.50	*1.42×10* ^−4^	5.57		*3.88×10* ^−7^	0.83		0.67
	Past 1 month	4.25	*1.42×10* ^−4^	4.17		*3.29×10* ^−5^	0.63		0.72
**Being disruptive in a social environment (eg, at school or elsewhere)**									
	Past ≥1 year	3.22	*1.56×10* ^−3^	4.36		*1.69×10* ^−5^	1.19		0.67
	Past 1 month to 1 year	3.87	*3.15×10* ^−4^	5.17		*6.98×10* ^−7^	1.72		0.55
	Past 1 month	3.77	*3.19×10* ^−4^	4.73		*3.53×10* ^−6^	1.78		0.55
**Attention problem**									
	Past ≥1 year	3.28	*1.34×10* ^−3^	4.72		*3.61×10* ^−6^	1.40		0.55
	Past 1 month to 1 year	2.87	*4.83×10* ^−3^	4.21		*3.00×10* ^−5^	1.60		0.55
	Past 1 month	2.18	*2.92×10* ^−2^	3.76		*1.70×10* ^−4^	1.40		0.55
**Breaking rules at school or elsewhere**									
	Past ≥1 year	3.45	*8.36×10* ^−4^	4.82		*2.53×10* ^−6^	1.03		0.67
	Past 1 month to 1 year	3.63	*4.84×10* ^−4^	4.97		*1.39×10* ^−6^	0.89		0.67
	Past 1 month	3.56	*5.84×10* ^−4^	5.37		*3.88×10* ^−7^	0.42		0.79

### MVLR: Using Demographics and Survey Questions to Model COVID-19 Likelihood

In brief, using MVLR with iterative resampling, we observed a model accuracy of 72% to 94% for an increased incidence of COVID-19 in participants with psychotic symptoms or VIDB >1 year ago (ie, before the pandemic).

Specifically, MVLR with iterative resampling focused on the responses from the time window of *past ≥1 year (long term)* for the 13 survey questions that significantly varied by COVID-19 status (Table 4 and Table 5). Responses to these 13 questions were used as *primary predictors* to model COVID-19 *test+* and *diagnosis* using MVLR. Given that no significant differences were observed in the survey question responses when split by responses to the COVID-19 *family* question, no attempt was made to model COVID-19 *family* using MVLR. The significant demographic variables of age, income, and education level were used as *covariates* in the model. To avoid overfitting of the model to the majority class (ie, participants in COVID-19 *test+* no and *diagnosis* no groups), the majority class was iteratively downsampled 1000 times, and the MVLR model was run each time. The average accuracy, SD of the accuracy, average RMSE, and average MAE across all iterations are reported. The null model with only the survey questions regarding psychosis with responses from >1 year ago (ie, Psych1-LT, Psych2-LT, Psych3-LT, and Psych4-LT) had an average accuracy of 72.08% for *test+* and 76.75% for *diagnosis*. The model with 4 survey questions as predictors along with demographic variables as covariates had an average accuracy of 85.45% for model *test+* and 89.43% for model *diagnosis* (Table 6). Similarly, the null model for 9 survey questions regarding VIDB with responses from more than a year ago (Disruption1-LT, Disruption2-LT..., Disruption9-LT) had average accuracies of 81.32% and 87.53% for *test+* and *diagnosis*, respectively. The 9 survey questions, along with covariates, had an average accuracy of 90.35% for the model *test+* and 94.39% for the model *diagnosis* (Table 6). Additional measures, including the SD of the accuracy, average RMSE, and average MAE for these models are also reported in Table 6.

**Table 6:** Multivariable logistic regression results^a^.

COVID-19 question (dependent variable)	Independent variables	Covariates	Accuracy, mean (SD)	RMSE^b^, mean (SD)	MAE^c^, mean (SD)
Test+	Psych1-LT, Psych2-LT, Psych3-LT, and Psych4-LT	—^d^	72.08 (3.80)	0.53 (0.04)	0.28 (0.04)
Test+	Psych1-LT, Psych2-LT, Psych3-LT, and Psych4-LT	Age Income Education level	85.45 (4.60)	0.38 (0.06)	0.15 (0.04)
Diagnosis	Psych1-LT, Psych2-LT, Psych3-LT, and Psych4-LT	—	76.75 (3.67)	0.48 (0.04)	0.23 (0.04)
Diagnosis	Psych1-LT, Psych2-LT, Psych3-LT, and Psych4-LT	Age Income Education level	89.43 (4.44)	0.32 (0.08)	0.11 (0.04)
Test+	Disruption1-LT, Disruption2-LT, Disruption3-LT, Disruption4-LT, Disruption5-LT, Disruption6-LT, Disruption7-LT, Disruption8-LT, and Disruption9-LT	—	81.32 (1.09)	0.43 (0.01)	0.19 (0.01)
Test+	Disruption1-LT, Disruption2-LT, Disruption3-LT, Disruption4-LT, Disruption5-LT, Disruption6-LT, Disruption7-LT, Disruption8-LT, and Disruption9-LT	Age Income Education level	90.35 (3.20)	0.31 (0.05)	0.10 (0.03)
Diagnosis	Disruption1-LT, Disruption2-LT, Disruption3-LT, Disruption4-LT, Disruption5-LT, Disruption6-LT, Disruption7-LT, Disruption8-LT, and Disruption9-LT	—	87.53 (1.15)	0.35 (0.02)	0.12 (0.01)
Diagnosis	Disruption1-LT, Disruption2-LT, Disruption3-LT, Disruption4-LT, Disruption5-LT, Disruption6-LT, Disruption7-LT, Disruption8-LT, and Disruption9-LT	Age Income Education level	94.39 (3.51)	0.22 (0.09)	0.06 (0.03)

^a^The table lists the dependent variable, independent variables, and covariates for each multivariable logistic regression model and reports the average and SD of accuracy, RMSE, and MAE.

^b^RMSE: root mean square error.

^c^MAE: mean absolute error.

^d^No covariates were included in the model.

## Discussion

### Principal Findings

In a small population sample, we assessed the frequency of self-reported VIDB and psychotic symptoms among people with or proximate to someone with a COVID-19 diagnosis and further tested the potential for discriminating who might become infected by COVID-19 based on a preceding history of these behaviors. For all 3 time windows, we found that participants who answered yes to having COVID-19 (*test+* and *diagnosis*) experienced VIDB and psychotic symptoms more frequently than those who answered no. No significant differences were observed between the yes or no distributions for those who had a close friend or family contract COVID-19 (ie, *family*). Using MVLR with iterative resampling to avoid model overfitting, we also found that participants who experienced psychotic symptoms and VIDB more frequently before the COVID-19 pandemic were more likely to subsequently contract COVID-19, suggesting that individuals experiencing psychotic symptoms and VIDB may (1) have comorbid conditions that increase their susceptibility to COVID-19 infection and (2) engage in riskier-than-average behaviors, potentially including nonadherence to COVID-19 precautions and engaging with other people.

The 3 demographic variables (age, income, and educational level) were significantly different when divided into yes or no distributions for COVID-19. Regarding age, COVID-19 was more prevalent in middle-aged individuals, with *yes* distributions for both COVID-19 infection questions having a median age of 37 years. These results contrast with some reports of COVID-19 incidence, where it was reported that older adults were more likely to be hospitalized because of COVID-19 [48], but support the results of other studies, in which younger to middle-aged people were more likely to contract COVID-19 [49]. It should be noted that this study only asked about the diagnosis or testing positive for COVID-19, which is different from hospitalization.

Annual household income for participants who answered yes to COVID-19 questions was more equally distributed across all income levels, with a peak at higher-income households (>US $75,000). In comparison, the income distribution of participants who answered no was skewed, with a higher concentration of participants in lower-income households. However, the participants who answered yes were evenly distributed across all income levels. This demonstrates that people who answered no for COVID-19 *test+* and *diagnosis* were predominately from lower-income households and that people who answered yes to *test+* and *diagnosis* were evenly distributed across all household income levels. This suggests that COVID-19 occurred in people with a wide range of income levels and did not preferentially occur in those living in lower- or higher-income households. These findings are in line with those reported in the study by Alsan et al [49] but are in contrast with those in the study by Baena-Diéz et al [50]. This study [50] found that people belonging to lower-income level households had higher incidence rates of contracting COVID-19 infection in Barcelona, Spain. Education had bimodal distributions for participants who answered yes to COVID-19 questions, with a larger peak at higher education levels. For participants who responded no, the distribution was skewed to the left, suggesting that education level did not predispose individuals to COVID-19.

Responses to survey questions about VIDB and psychotic symptoms exhibited higher scores for participants who answered yes for COVID-19 than for people who responded no. This observation showed similar statistical effects regardless of the time window assessed (ie, in the past 1 month, 1 month to 1 year ago, and >1 year ago; Tables 4 and 5). Given these findings for all time windows in this specific study, including those before the pandemic, our observation of a higher frequency of psychotic episodes is less likely to be a direct consequence of COVID-19, which has been linked to cognitive and attention deficits, brain fog, and anxiety [2]. For the same reason, our findings are less likely to be because of higher doses of steroid treatment, which is known to complicate or trigger psychosis [51]. It should be noted that there have been multiple reports of subacute onset of psychotic episodes in healthy individuals with no prior history of mental illness after treatment for COVID-19 [52-55]. These psychotic episodes also led to aggressive, violent, and disruptive behavior and a preponderance of not following rules [53,55]. The psychological impacts of quarantine, temporary loss of employment, lack of livelihood, and financial insecurity have also been shown to trigger violent and disruptive behavior [8,56]. Owing to feelings of frustration and agitation associated with COVID-19, pandemic-related stressors may manifest as aggression and violence toward household members [57-59]. Fear surrounding the COVID-19 pandemic has also been associated with aggressive behavior on the web [60]. Although this is consistent with our results where participants who contracted SARS-CoV-2 reported higher incidences of psychotic symptoms and VIDB in the past 1 month and 1 month to 1 year, our observation of these symptoms, ideation, and behavior before the pandemic for these participants suggests that COVID-19 infection was not causal in the presentation of VIDB or psychotic symptoms. Our study did not find associations that were directional, namely, whether COVID-19 infection increases the likelihood of psychosis and VIDB or whether psychotic symptoms and VIDB increase the possibility of COVID-19 infection. Other viral infections, including influenza and HIV, have led to similar hypotheses [26-34]. Recently, a study by Douaud et al [61] observed a greater reduction in overall brain size, reduction in gray matter thickness and tissue contrast in the orbitofrontal cortex and parahippocampal gyrus, and changes in markers of tissue damage in regions functionally connected to the primary olfactory cortex in people who tested positive for infection with SARS-CoV-2. Similar brain regions are known to be affected in the same way by psychosis and VIDB [62-66].

To assess whether self-reported VIDB or psychotic symptoms that occurred >1 year ago (ie, before the pandemic) modeled that individuals subsequently had COVID-19 infection, MVLR was performed with iterative downsampling of the majority class to reduce the possibility of model overfitting (Table 6). The demographic variables of age, income, and education level were used as covariates. The full model was not confounded by covariates as the highest accuracy without covariates (ie, null model) was 87.5%, whereas the highest accuracy for the full model was 94.4%. Across all the models, the accuracy of modeling COVID-19 by *diagnosis* was higher than that for *test+*. This might be because of the likelihood of false positives from COVID-19 testing and the fact that COVID-19 testing is an inherent component of clinical diagnosis, making clinical diagnosis more informed. There was also a lag at the beginning of the pandemic before testing was widely available. Overall, the highest accuracy for modeling COVID-19 infection from psychotic symptoms from >1 year ago was 89.4%, and for VIDB, it was 94.4%. These results may reflect comorbid conditions that increase susceptibility to COVID-19 infection or reflect engagement in riskier-than-average behaviors, potentially including nonadherence to COVID-19 precautions, as has been observed with other viral infections [30-32]. Conversely, there is another hypothesis that people with mental health conditions become more antisocial and are thus more distant from other people, potentially resulting in a reduced viral spread. Further work is needed to determine whether psychotic symptoms or VIDB increase the risk for, and the spread of, COVID-19.

This study has several limitations. First, this study involved a small sample size of participants responding yes to *test+* (36/366, 9.8%) and *diagnosis* (34/366, 9.3%) in our cohort. Although the percentage of studied participants infected with COVID-19 herein was consistent with the population estimates of COVID-19 in the United States at the time of data collection, future work needs to assess larger population samples. Another limitation is the bias arising from the false positives of self-reported COVID-19 test positives. It should be noted that the survey did not collect data when the respondents were infected with COVID-19 in the previous year; this is a limitation in estimating the precise time frame for an increase in behavioral symptoms associated with COVID-19. Recall bias is a well-known caveat for any study that uses survey methods [67]. Furthermore, it should also be noted that MVLR results do not represent true predictions, which would require adequately sized training and test sets, along with cross-validation of prediction outcomes. Future work will need to use larger sample sizes and perform better predictive analyses with more sophisticated machine learning algorithms. These caveats aside, it must be noted that this study used iterative resampling to overcome a major confounder that is common in current machine learning papers with class imbalance in smaller data sets—model overfitting—supporting the generalizability of these results.

### Conclusions

This preliminary study examined a population sample collected at the end of February 2021 and the beginning of March 2021 and found that people who reported a test and clinician diagnosis of COVID-19 also reported higher frequencies of VIDB or psychotic symptoms across multiple time windows (the past 1 month, 1 month to 1 year ago, and >1 year ago), including those before the pandemic started. We also found that participants who experienced VIDB or psychotic symptoms >1 year ago (ie, before the pandemic) were more likely to contract SARS-CoV-2 or be diagnosed with COVID-19. A greater understanding of how COVID-19 may trigger these mental health issues is needed, given other literature on viral, carcinogenic, and toxic effects in changing mental status, including triggering psychotic symptoms and disruptive behavior [26-29,33,34,68-71]. It is also of utmost importance to understand which vulnerable populations are at a greater risk of contracting and spreading the virus to curb the viral spread, which includes vulnerable populations related to age, sex, and household income.

## Appendix

Multimedia Appendix 1 Survey questions and box plots of survey questions for yes or no distributions based on COVID-19 questions.

## Abbreviations

MAE: mean absolute error
MGH SQ: Massachusetts General Hospital Subjective Question
MVLR: multivariable logistic regression
RMSE: root mean square error
VIDB: violent ideation and disruptive behavior
