# Supplementary material for: The Prevalence of Psychotic Symptoms, Violent Ideation, and Disruptive Behavior in a Population With SARS-CoV-2 Infection: Preliminary Study
Source: JMIR Form Res. 2022 Aug 16;6(8):e36444. doi: 10.2196/36444 (PMC9384857; doi:10.2196/36444)
Supplement: Multimedia Appendix 1 [file formative_v6i8e36444_app1.docx]

**Supplemental Material**

**Methods**

**Survey Questions and scoring**

Out of the larger survey the thirteen questions used in this study are as follows:

**Please rate how often you have experienced each of the following in the past year and in the past one month:**

1 2 3 4 5 6 7

Never Rarely Sometimes Often Always

Hallucinations/Hearing voices others cannot

Past 1 year or more:

Past 1 month to 1 year:

Past 1 month:

Hallucinations/Seeing things others cannot

Past 1 year or more:

Past 1 month to 1 year:

Past 1 month:

Worries that others are out to get you, or to get people close to you (these might resemble paranoia)

Past 1 year or more:

Past 1 month to 1 year:

Past 1 month:

Having one or more unique beliefs or impressions that are strong despite being contradicted by others (these might resemble delusions)

Past 1 year or more:

Past 1 month to 1 year:

Past 1 month:

Wanting to hurt others

Past 1 year or more:

Past 1 month to 1 year:

Past 1 month:

Prior attempts at hurting others

Past 1 year or more:

Past 1 month to 1 year:

Past 1 month:

Having a plan for not hurting others when these feelings arise

Past 1 year or more:

Past 1 month to 1 year:

Past 1 month:

Prior attempts at hurting insects or small animals

Past 1 year or more:

Past 1 month to 1 year:

Past 1 month:

Intrusive thoughts that lead you to repetitive actions

Past 1 year or more:

Past 1 month to 1 year:

Past 1 month:

Desire to start fires

Past 1 year or more:

Past 1 month to 1 year:

Past 1 month:

Being disruptive in a social environment (e.g., at school or elsewhere)

Past 1 year or more:

Past 1 month to 1 year:

Past 1 month:

Attention problems

Past 1 year or more:

Past 1 month to 1 year:

Past 1 month:

Breaking rules at school or elsewhere

Past 1 year or more:

Past 1 month to 1 year:

Past 1 month:

**Supplemental Figure 1**

**
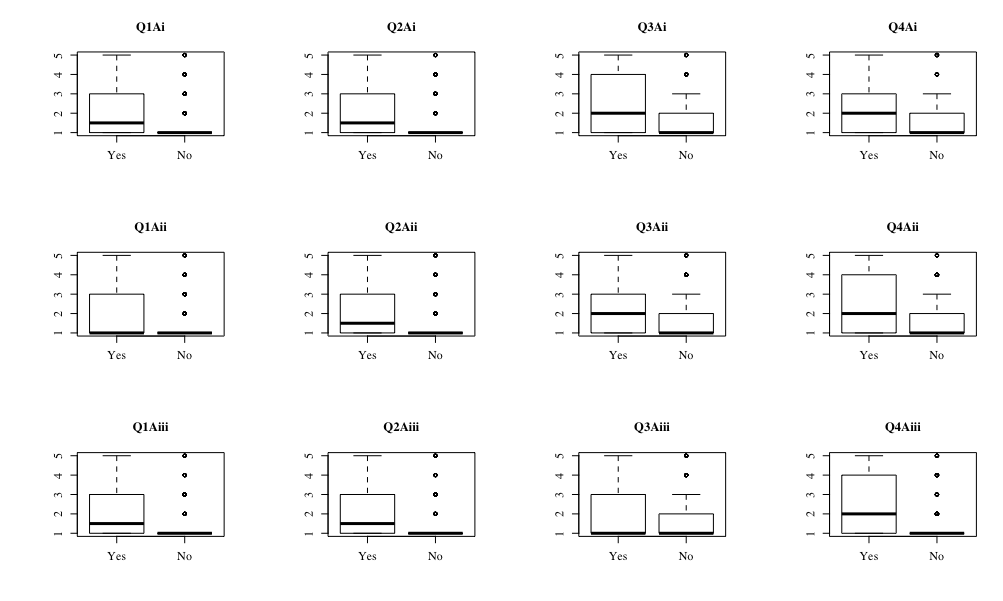
(A)**

**(B)**

**
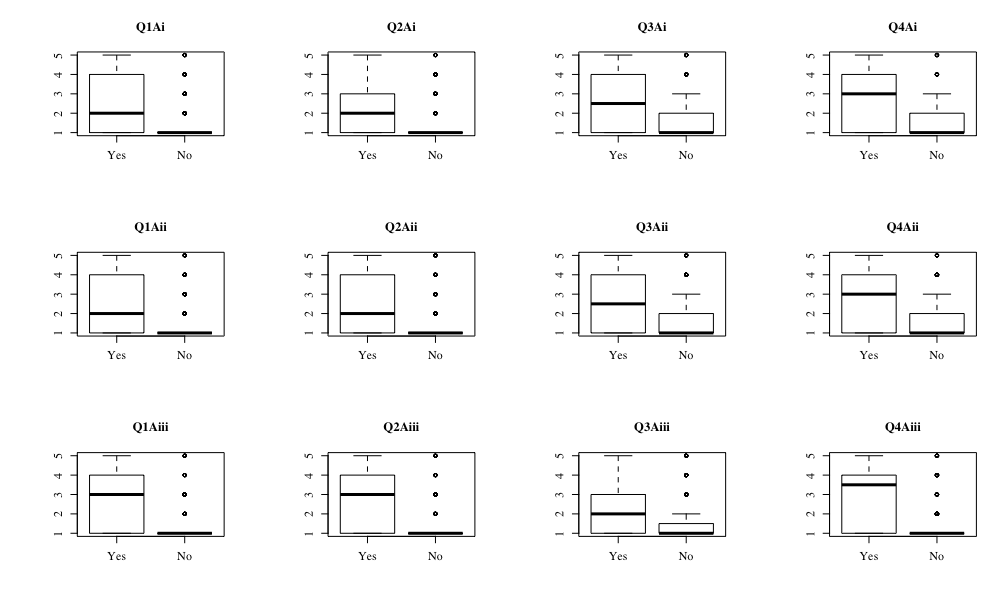
**

**(C)**

**
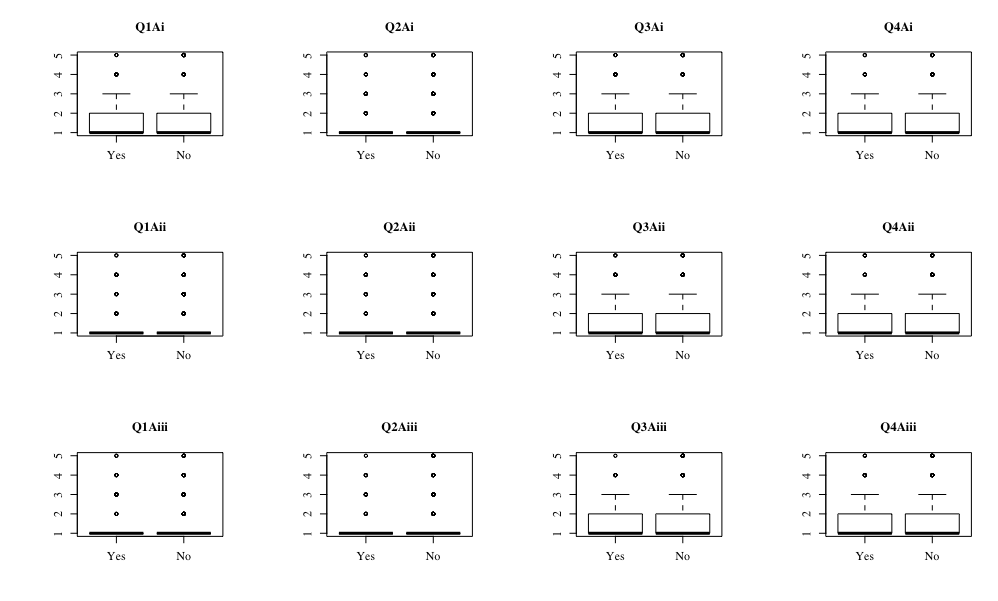
**

**Supplemental Figure 1.** Box plots for yes/no distributions based on COVID-19 questions **(A)** *test+*, **(B)** *diagnosis* and **(C)** *family* for survey questions (**1A-4A)** related to psychosis.

**Supplemental Figure 2.**

**(A)**

**
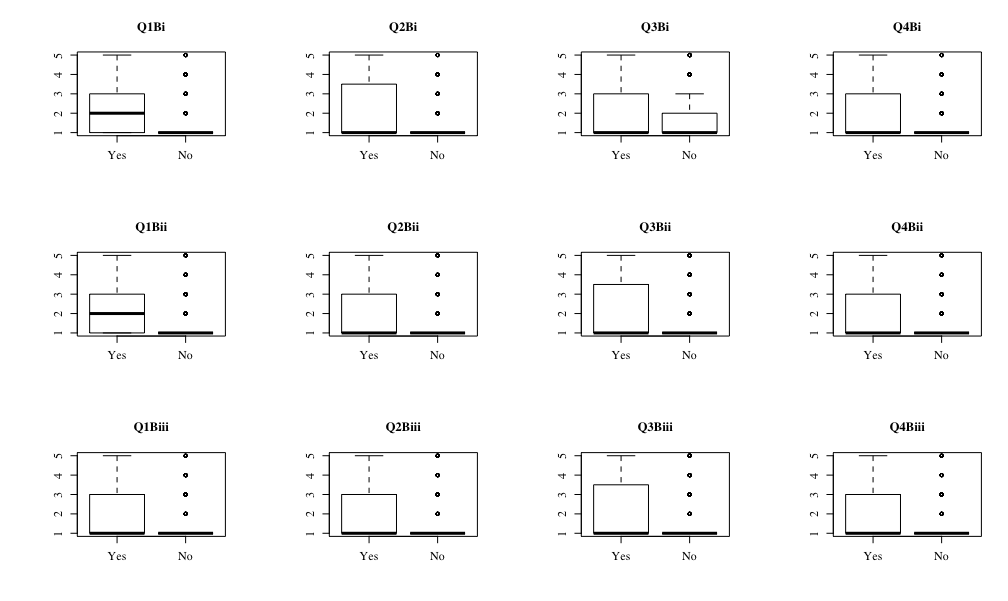
**

**
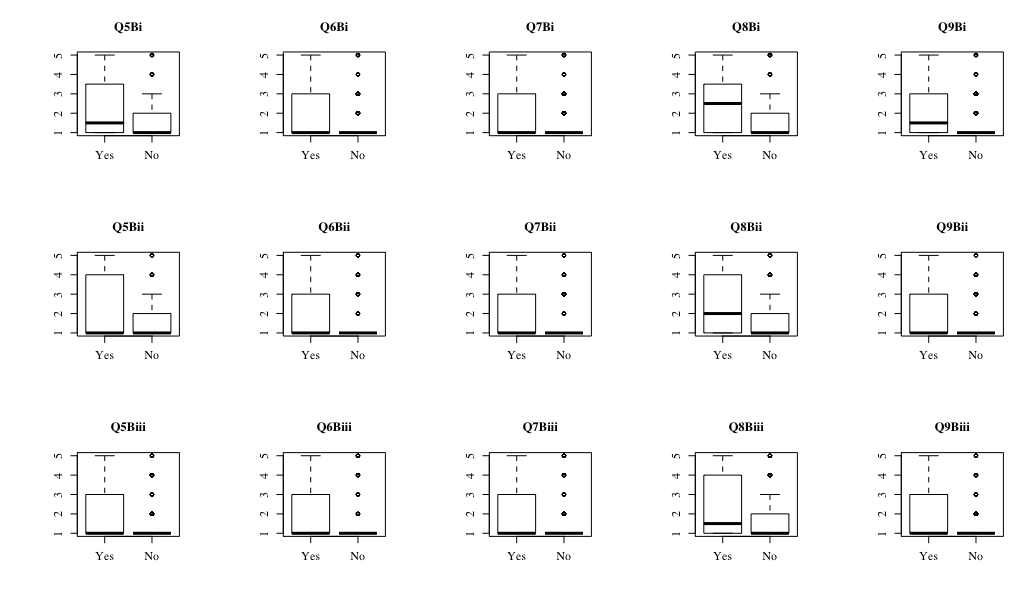
**

**(B)**

**
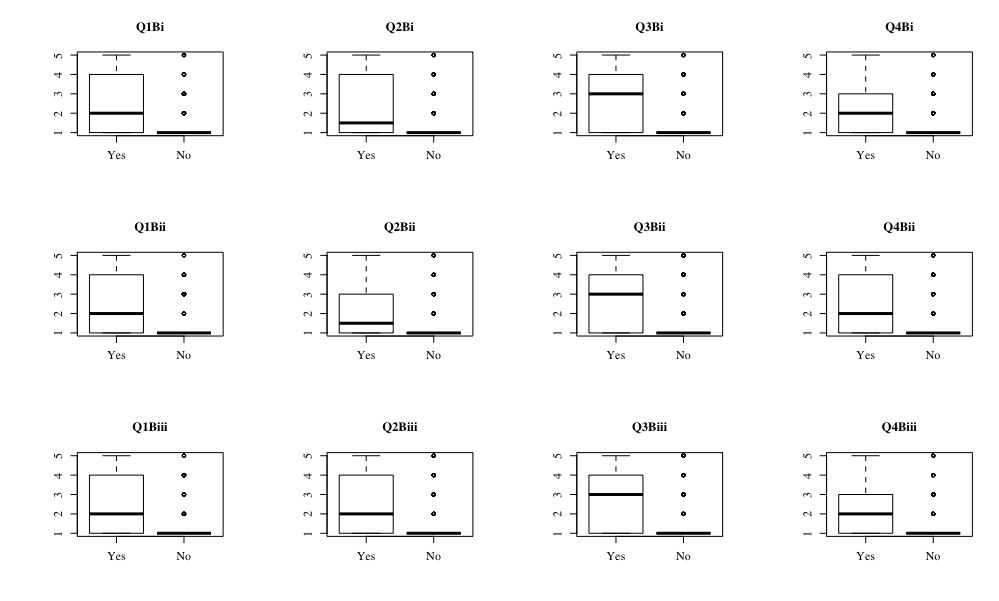
**

**
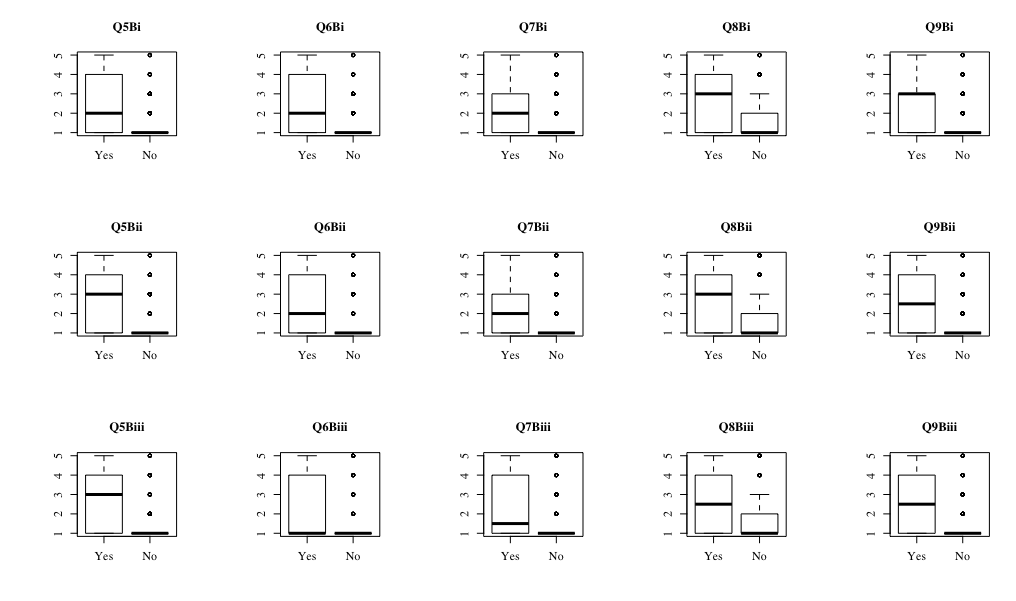
**

**(C)**

**
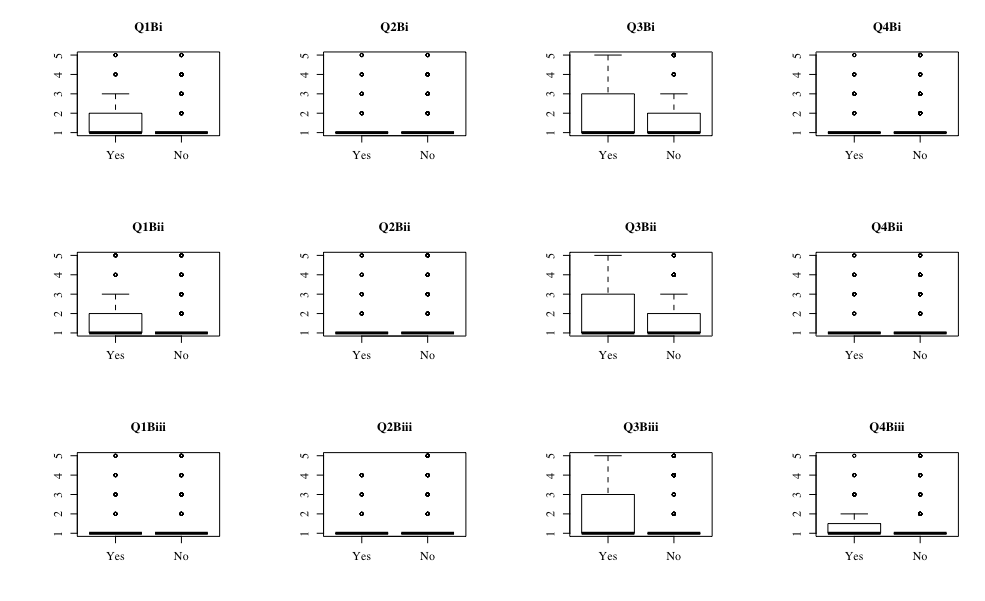
**

**
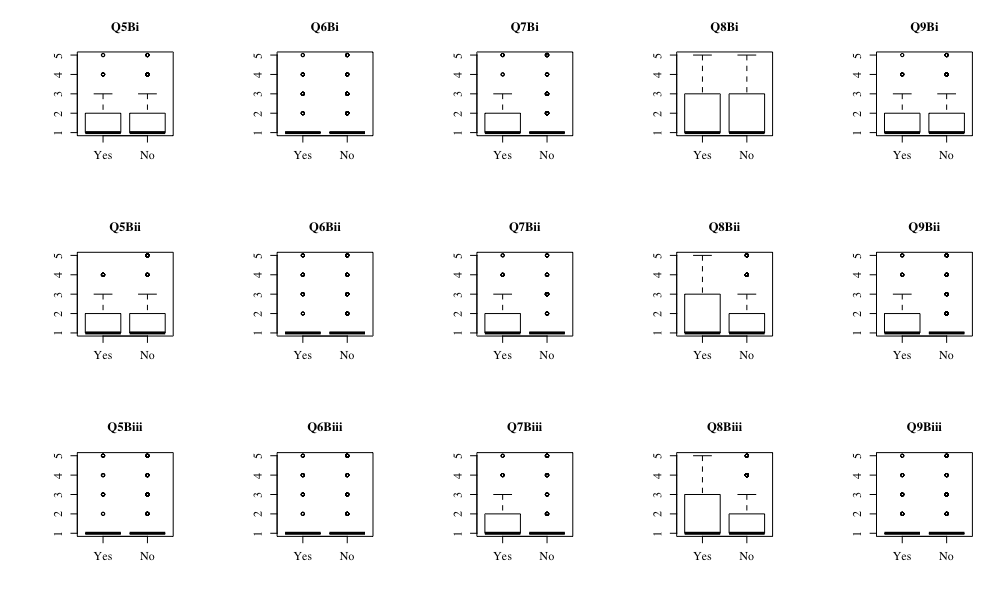
**

**Supplemental Figure 2.** Box plots for yes/no distributions based on COVID-19 questions **(A)** *test+*, **(B)** *diagnosis* and **(C)** *family* for survey questions (**1B-9B)** related to violent ideation and disruptive behavior.
